# Neutrophil Gelatinase-Associated Lipocalin (NGAL), Pro-Matrix Metalloproteinase-9 (pro-MMP-9) and Their Complex Pro-MMP-9/NGAL in Leukaemias

**DOI:** 10.3390/cancers6020796

**Published:** 2014-04-04

**Authors:** Sandrine Bouchet, Brigitte Bauvois

**Affiliations:** INSERM U1138, Université Pierre et Marie Curie, Université Paris-Descartes, Centre de Recherche des Cordeliers, Paris 75006, France; E-Mail: sandrine.bouchet@crc.jussieu.fr

**Keywords:** hematologic malignancy, gelatinase, cancer, signalling, cell surface binding

## Abstract

Matrix metalloproteinase (MMP)-9 and neutrophil gelatinase-associated lipocalin (NGAL) have gained attention as cancer biomarkers. The inactive zymogen form of MMP-9 (pro-MMP-9) also exists as a disulphide-linked heterodimer bound to NGAL in humans. Leukaemias represent a heterogeneous group of neoplasms, which vary in their clinical behavior and pathophysiology. In this review, we summarize the current literature on the expression profiles of pro-MMP-9 and NGAL as prognostic factors in leukaemias. We also report the expression of the pro-MMP-9/NGAL complex in these diseases. We discuss the roles of (pro)-MMP-9 (active and latent forms) and NGAL in tumour development, and evaluate the mechanisms by which pro-MMP-9/NGAL may influence the actions of (pro)-MMP-9 and NGAL in cancer. Emerging knowledge about the coexpression and the biology of (pro)-MMP-9, NGAL and their complex in cancer including leukaemia may improve treatment outcomes.

## 1. Introduction

Of the matrix metalloproteinases (MMPs) thought to be involved in cancer, attention has focused on MMP-9 because of its deregulated expression in cancer and its association with tumours’ invasive potential [1,2]. In most cancers, MMP-9 is found expressed as pro-MMP-9 which is the inactive zymogen form of the enzyme. Neutrophil gelatinase-associated lipocalin (NGAL) was first purified from human neutrophils because of its ability to fix pro-MMP-9 by forming a disulphide-linked heterodimer [3,4]. Both NGAL and MMP-9 (active and latent) have already emerged as useful biomarkers in a wide array of malignant diseases including breast, brain, ovarian, pancreas, colorectal, bladder, prostate and lung and skin cancers [2,5,6,7,8]. On-going studies are investigating the value of the pro-MMP-9/NGAL complex as a marker of disease status in cancer. For example, the levels of pro-MMP-9/NGAL can be detected in tissues, urine and blood in breast, brain and gastric tumours and are significantly correlated with disease severity and poor survival [9,10].

Leukaemias are clonal disorders resulting from the neoplastic transformation of hematopoietic progenitor cells, associated with abnormal tumour cell growth, survival and dissemination from the bone marrow into blood and peripheral lymphoid tissues. The deregulated expression of pro-MMP-9 is observed in leukaemias [11,12,13,14]. However there are sparse data on the expression patterns of NGAL and/or the pro-MMP-9/NGAL complex in these diseases. This review is aimed at (i) providing an overview of the current literature on the expression profiles of pro-MMP-9, NGAL and their complex in leukaemias and (ii) highlighting the recent advances in understanding the roles of (pro)-MMP-9, NGAL and pro-MMP-9/NGAL in cancer including leukaemia.

## 2. Introducing (Pro)-MMP-9, NGAL and the Pro-MMP-9/NGAL Complex

Functional and structural components of MMP-9 include a hydrophobic signal peptide for secretion, a propeptide domain for enzyme latency, a catalytic domain with a highly conserved zinc-binding site, a collagen-binding domain within its catalytic domain and a hemopexin-like *C*-terminal domain (PEX) linked to the catalytic domain via a flexible O-glycosylated domain [1,2]. The enzyme is secreted as an inactive zymogen (pro-MMP-9, 92 kDa), with cleavage of the propeptide domain yielding the active MMP-9 (82 kDa). Plasmin, trypsin-2, MMP-2, MMP-13, MMP-3, serine elastase and kalikrein are amongst the many proteolytic activators of pro-MMP-9 [1,2]. The PEX domain is a four-bladed propeller structure within which each blade consists of four antiparallel β-sheets and one α-helix [2]. It contains three cysteine residues (at positions 516, 674 and 704), with one disulphide bond bridging Cys-516 in blade I and Cys-704 in blade IV [15]. Various soluble proteins are found to be bound (covalently or non-covalently) to PEX, including tissue inhibitor of metalloproteinase (TIMP)-1 and TIMP-3, extracellular matrix components, β-hematin, NGAL and pro-MMP-9 itself [1,2,16]. 

Neutrophil gelatinase-associated lipocalin is a secreted 25 kDa protein which exhibits a single, eight-stranded antiparallel β-barrel surrounding a central pocket that is capable of binding low-molecular-weight ligands (such as *N*-formylmethionyl-leucyl-phenylalanine, retinoids, steroids and fatty acids) and of capturing siderophores (such as bacterial enterochelin and mammalian endogenous catechols) that bind iron with high affinity [7]. NGAL also exists as: (i) a 46 kDa disulphide-linked homodimer; (ii) a homotrimer of 70 kDa; and (iii) a 130 kDa disulphide-linked heterodimer bound to pro-MMP-9 [7]. The Cys-87 in NGAL forms a disulphide bond with an as yet unidentified cysteine residue in MMP-9’s PEX domain [7].

## 3. Pro-MMP-9, NGAL and Pro-MMP-9/NGAL as Leukaemia Biomarkers

In the normal and tumoral hematopoietic compartment, MMP-9 is released as pro-MMP-9. As determined by ELISAs, detectable levels of MMP-9 (total; 92 kDa pro and 82 kDa active forms) (median level 67 ng/mL), NGAL (median level 72 ng/mL) and pro-MMP-9/NGAL (median level 40 ng/mL) are observed in the systemic circulation of healthy subjects [4,17,18,19]. Normal immature (CD34^+^) bone marrow progenitor cells express NGAL [20] but not (pro)MMP-9 [21]. During maturation of granulocyte precursors in the bone marrow, NGAL is synthesized almost exclusively by myelocytes and metamyelocytes [22]. NGAL is also expressed in human erythroid cells [20]. Expression of NGAL, pro-MMP-9 and the 130 kDa pro-MMP-9/NGAL complex is observed in activated monocytes and neutrophils [4,20,23]. Resting T and B lymphocytes express the mRNAs for NGAL and MMP-9 [20,24,25]. The production of pro-MMP-9 protein appears to be dependent on the activation status of T- and B-cells and to be regulated by cytokines [24,26]. Although the transcription factor NF-κB is expressed in an inactive state in normal leukocytes, leukaemia cells express activated NF-κB [27]. The NF-κB signalling pathway regulates the transcription of both MMP-9 and NGAL [7,15]. This may explain the abnormal expression of pro-MMP-9 and NGAL in leukaemias.

### 3.1. MMP-9 as a Prognostic Factor in Chronic Lymphocytic Leukaemia (CLL)

**Figure 1:**
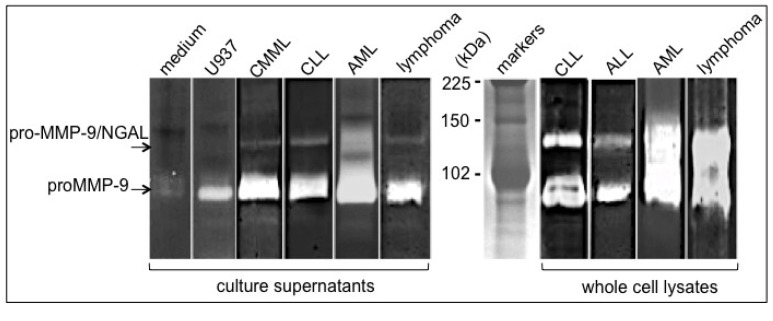
Detection of pro-MMP-9 and pro-MMP-9/NGAL levels in hematopoietic malignant cells. Blood samples were obtained from patients with chronic myelomonocytic leukemia/CMML, chronic lymphocytic leukemia/CLL, acute lymphoid leukemia/B-ALL, acute myeloid leukemia/AML and Burkitt’s lymphoma. Peripheral blood mononuclear cells were separated by Ficoll-Hypaque density gradient centrifugation, washed twice in PBS, lysed or cultured as described in [38]. Whole cell lysates were obtained by lysing freshly isolated cells in M-PER buffer (4 × 10^6^ cells/30 μL) supplemented with protease and phosphatase inhibitor cocktails as described in [38]. As a positive control for pro-MMP-9 release, U937 cells (ATCC CRL-1593.2), cultured as described in [39] were stimulated with 100 U/mL recombinant TNF-α for 48 h (R&D). The 48 h-culture supernatants from U937 cells (2 × 10^5^/mL) and primary leukaemia cells (2 × 10^6^/mL) were harvested by centrifugation and frozen until zymography. Control medium alone was incubated under the same conditions. Analysis of (pro)MMP-9 and NGAL presence in culture supernatants (30 μL) and whole cell lysates (30 μL) was carried out in 7.5% (w/v) SDS-PAGE containing 0.1% gelatin (w/v) as described elsewhere [18]. Zymograms showed two major bands of 130 kDa and 92 kDa corresponding respectively to pro-MMP-9/NGAL and pro-MMP-9. The sizes were determined by interpolation from a standard curve of Rf values of known molecular weight markers.

Chronic lymphocytic leukaemia is characterized by accumulation in the blood of clonal expansions of CD5^+^/CD23^+^ B lymphocytes [28]. The accumulated leukemic cells (which are mostly quiescent) result mainly from their inability to develop an apoptotic program—although proliferating pools are found in the bone marrow and lymph nodes [28]. In contrast to resting B lymphocytes, CLL cells (stage A according to Binet’s classification) synthesize and secrete pro-MMP-9 [18,29]. Accordingly, serum MMP-9 concentrations are significantly higher in untreated early-CLL patients (stage A) than in healthy controls [18,30,31] and decrease to near-control levels in patients in remission [31]. Moreover, higher levels of intracellular MMP-9 are associated with advanced (stage C) disease and with poor overall survival [29]. These MMP-9 findings could help to screen patients with CLL to determine their risk of disease progression. The pro-MMP-9/NGAL complex is found released by CLL blood cells and expressed in CLL blood cell lysates [29] (Figure 1), strongly suggesting that NGAL and pro-MMP-9 could form a complex within the cell prior to secretion.

### 3.2. MMP-9 as a Prognostic Factor in Acute Lymphoblastic Leukaemia (ALL)

Acute lymphoblastic leukaemia (ALL) is a heterogeneous disease that includes B and T-ALL cancers. B-ALL is characterized by an accumulation of early B blood cells, which can infiltrate lymph nodes, liver, spleen and lung [32]. T-ALL cells arise from the malignant transformation of hematopoietic progenitors primed for T cell development [32]. Although T-ALL develops mostly in the thymus, it tends to spread throughout the body (including infiltration of the bone marrow and the central nervous system) [32]. Blood mononuclear cells from T-ALL and B-ALL patients express pro-MMP-9 [13,14,33,34]. Our group reported pro-MMP-9/NGAL expression in B-ALL cells (Figure 1). At diagnosis, plasma and serum MMP-9 levels are lower in the T- and B-ALL patients than in the normal controls [31,35,36,37]. However, a significant elevation of plasma MMP-9 is observed in T-ALL patients with malignant cell infiltration [34]. Moreover, elevated secretion of pro-MMP-9 by B-ALL blood leukocytes is found associated with a lower overall survival rate [14]. These data suggest that MMP-9 may act as a prognostic marker for B- and T-ALL progression.

### 3.3. Link between NGAL and BCR-ABL in Chronic Myeloid Leukaemia (CML)

Chronic myeloid leukaemia is a clonal myeloproliferative disorder that originates from a pluripotent stem cell expressing the Ph chromosome (t(9;22) chromosomal translocation) with the constitutively active BCR-ABL fusion gene, which leads to the production of the p210 BCR-ABL protein [40]. During the progression of CML, leukemic cells gradually replace normal bone marrow mononuclear cells and overpopulate the spleen and liver, resulting in anaemia and a high number of white blood cells in the peripheral blood. Hyperproliferation of white blood cells is the direct result of the constitutive tyrosine kinase activity of p210 BCR-ABL which activates major signal transduction pathways [40]. Inhibition of this kinase with the drug imatinib (approved by the U.S. Food and Drug Administration in 2001) leads to alleviation of hyperproliferative symptoms [41,42]. Bone marrow and blood mononuclear cells from CML patients express pro-MMP-9 and NGAL proteins [12,43,44,45]. NGAL expression correlates with that of BCR-ABL [46,47]. Serum/plasma levels of MMP-9 and NGAL are significantly higher in CML patients than in healthy individuals [36,46,47,48]. If CML patients achieve complete molecular remission after imatinib therapy, NGAL serum levels fall and are significantly lower than the disease-state value [47,48]. These findings suggest a potential role of NGAL in monitoring the efficacy of the treatment of CML.

### 3.4. MMP-9 and NGAL as Markers for Prognosis in Acute Myeloid Leukaemia (AML)

Acute myeloid leukaemia (AML) is a clinically and genetically heterogeneous haematopoietic cancer characterized by the clonal expansion and accumulation of immature myeloid precursors in the bone marrow and blood [49,50]. Distinct AML subfamilies (French/American/British (FAB) subtypes) are defined by the development stage at which the cells are arrested [49,50]. AML cells disseminate from the bone marrow into peripheral tissues. Most patients with AML have poor rates of survival associated with a plethora of mutations such as internal tandem duplication (ITD) in the FLT3 gene [50]. Blood and bone marrow AML blasts express and secrete pro-MMP-9 (independently of their FAB subtype) [21,51]. The pro-MMP-9/NGAL complex is found in AML cells that contain very large amounts of pro-MMP-9 (Figure 1). Serum levels of MMP-9 are markedly lower in AML patients than in healthy individuals [36]. Accordingly, bone marrow pro-MMP-9 levels are also significantly lower in AML patients than in normal controls; the levels recover to normal values following complete remission and decline again at relapse [35]. Moreover, bone marrow MMP-9 levels are significantly higher in patients with extramedullary infiltration than in patients without infiltration - suggesting that MMP-9 production by leukemic cells might contribute to the latter’s dissemination from the bone marrow [52]. Similarly, NGAL expression in the bone marrow is lower in AML patients than in normal controls [53]. Likewise, NGAL expression increased in patients achieving complete remission and falls in patients with refractory disease [53]. In addition, a combination of FLT3-ID mutation status and high NGAL levels is predictive of the best survival rates in patients with AML [53]. These data suggest that MMP-9 and NGAL might be surrogate markers of disease status in patients with AML.

## 4. Roles of MMP-9, Pro-MMP-9 and NGAL in Cancer

Extensive research of MMP-9 and NGAL has demonstrated their involvement in fundamental biological processes including inflammation and cancer [7,9,10,15,54]. Moreover, inflammation can affect tumor development and progression [55]. Indeed, the tumor microenvironment contains immune and inflammatory cells in addition to the cancer cells and their surrounding stroma (which consists of fibroblasts, endothelial cells, pericytes and mesenchymal cells) [55,56]. These diverse cells produce a wide variety of inflammatory cytokines, chemokines, reactive oxygen species and secreted proteases (such as MMP-9), which in autocrine and paracrine manners control tumor progression [55,56,57]. These evidences further support the multiple roles of MMP-9 observed in cancer and summarized below.

### 4.1. MMP-9 and pro-MMP-9

**Figure 2:**
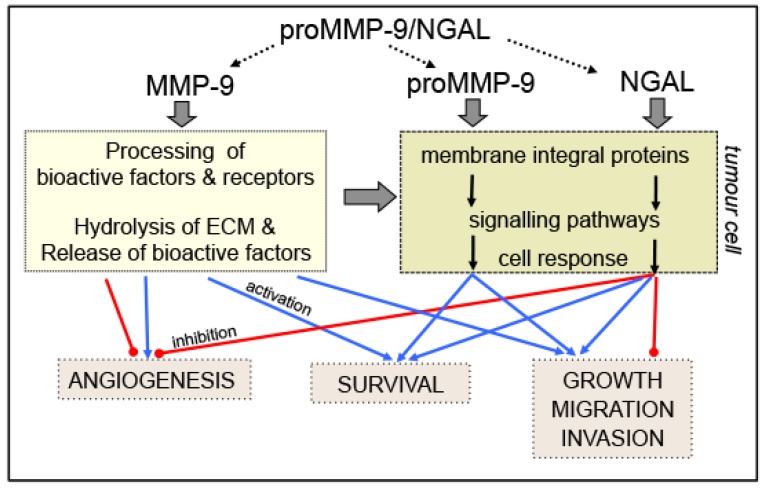
Schematic diagram of the roles of (pro)MMP-9, NGAL and pro-MMP-9/NGAL in cancer. Active MMP-9 degrades structural components within the ECM, facilitating tumor cell invasion and metastasis and thus releasing bioactive factors (growth factors, cytokines and angiogenic factors) embedded in the ECM. MMP-9 also generates angiogenesis inhibitors, such as endostatin and tumstatin. MMP-9 processes and activates or inactivates signalling molecules (cytokines, chemokines, growth factors, receptors) that target tumor cells (cell growth, survival, migration, invasion and metastasis) and surrounding endothelial cells (tumour-associated angiogenesis). NGAL (whether bound to siderophore/iron or not) and pro-MMP-9 bind to integral membrane proteins on tumour cells leading to pro- (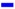) or anti- (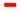) tumour effects on growth, survival, migration, invasion and angiogenesis. The possible actions of the pro-MMP-9/NGAL complex on cell events remain to be identified (**.....**).

The role of MMP-9 through its hydrolytic activity has been discussed in excellent reviews [1,2,15]. By cleaving many different targets (extracellular matrix, cytokines, growth factors, chemokines, growth factor receptors), active MMP-9 releases or generates bioactive molecules that in turn bind to specific receptors known to regulate key signalling pathways associated with cell growth, migration, invasion and angiogenesis [1,2,15] (Figure 2). For example, MMP-9 can release factors such as vascular endothelial growth factor (VEGF), transforming growth factor (TGF)-β1 and fibroblast growth factor (FGF)-2 sequestered in the extracellular matrix which stimulate tumour associated-endothelial cells and thus promote angiogenesis and tumor growth. In contrast, tumstatin and endostatin generated by the MMP-9-mediated proteolysis of type IV collagen and type XVIII collagen, respectively, are active inhibitors of angiogenesis. Moreover, MMP-9 sheds and activates pro-tumour necrosis factor (TNF)-α, proTGF-β1 and Kit-ligand which are intimately involved in the regulation of cell growth and angiogenesis. MMP-9 suppresses the proliferation of T lymphocytes through disruption of the IL-2R signalling that may constitute a mechanism of cancer-mediated immunosuppression [58]. By cleaving β-dystroglycan, MMP-9 (in concert with MMP-2) allows the entry of leukocytes into the outer parenchymal barrier, that may facilitate leukocyte infiltration into the CNS [59]. Finally, MMP-9 generates either inactivated chemokine fragments (e.g., growth-regulated protein (GRO)-α, platelet factor (PF)-4, stromal-cell derived factor (SDF)-1, monokine induced by interferon-γ (MIG)) or truncated chemokines with enhanced activity (interleukin (IL)-8, IFN-γ-induced T cell-activated chemokine (I-TAC)). The MMP-mediated proteolysis of chemokines might have direct consequences on tumor growth (e.g., I-TAC), migration (e.g., SDF-1) and angiogenesis (e.g., IL-8, PF-4, MIG and SDF-1) (reviewed in [1,2,15]).

Several research groups have shown that (pro)-MMP-9 interacts with the cell surfaces of leukocytes and epithelial and endothelial cells by binding to various integral membrane proteins such as integrins (αVβ/αβ1/αβ2), CD44, Ku protein and the low-density lipoprotein receptor-related proteins (LRP-1 and LRP-2) [60,61,62]. In chronic lymphocytic leukaemia (CLL) cells, CD44, integrin α4 (also known as CD49d) and pro-MMP-9 are physically linked to CD38 in a supramolecular cell surface complex [63]. There is now growing evidence pointing the ability of pro-MMP-9 to directly activate classical signalling pathways involved in cell growth, survival migration and angiogenesis [62] (Figure 2). For example, the binding of pro-MMP-9 to αMβ2 and CD44 induces the migration of monocytes and dendritic cells [64]. Similarly, the binding of pro-MMP-9 to the integrins αLβ2 and αMβ2 integrins induces the migration of human acute myeloid leukaemia (AML) cell lines and tumor-associated neutrophils [65]. The binding of pro-MMP-9 to its docking receptors α4β1 integrin and CD44 induces an intracellular signalling pathway that favours the growth and survival of CLL primary blood cells [66]. This pathway consists of Lyn kinase activation, STAT3 phosphorylation and activation of the pro-survival protein Mcl-1 (a member of the Bcl-2 family) [66].

### 4.2. NGAL

Human NGAL exhibits little similarity to the mouse homologue lipocalin-2 (Lcn-2) (62%) and contains an unpaired cysteine that can form the MMP-9/NGAL complex [7]. In contrast, the complex is not found in mice since Lcn-2 lacks the cysteine prerequisite for this binding [7]. These facts are crucial in the analysis of the attributed roles of NGAL (free or bound to MMP-9) in humans which might be distinct from that of Lcn-2 in mice. Therefore, we summarize here what is currently known of the biological activities of NGAL in the human system.

Like pro-MMP-9, NGAL is shown to interact as ligand with integral membrane proteins and this may induce a receptor-mediated effect on signalling pathways involved in biological events (Figure 2). So far, two cell surface receptors have been identified for NGAL, *i.e.*, LRP-2 (also known as megalin) and the solute carrier SLC22A17 (also known as 24p3R) [67,68,69]. Both of these receptors are able to bind NGAL alone or bound to a siderophore and iron [7].

NGAL is a recognized anti-bacterial factor of natural immunity through its ability to capture siderophores causing iron depletion and blocking bacterial cell growth [10]. Mounting evidence points towards growth factor effects of NGAL that modulate major cellular processes associated to tumoral development [7,9,70] (Figure 2). NGAL appears to exhibit either pro- or anti-tumour effects, depending on the type of cancer in question. NGAL facilitates the survival of human lung and breast carcinoma cells and can provide protection from the apoptosis induced by phosphoinositide-dependent kinase (PDK)-1 inhibitors [71]. NGAL increases the motility and invasion of human colon carcinoma cell lines by affecting the subcellular localization of E-cadherin and Rac1 (one of the Rho small GTPases) through an iron-dependent mechanism [72]. These data are consistent with those of Nuntagowat *et al*. where NGAL silencing suppresses human cell cholangiocarcinoma migration and invasion [73]. NGAL overexpression in human breast cancer cells leads to increased breast tumor proliferation [74]. Paradoxically, NGAL’s inhibition of the proliferation and invasion of human hepatocellular carcinoma cells is associated with the blockade of the c-Jun *N*-terminal kinase (JNK) and phosphoinositide 3-kinase (PI3)/AKT signalling pathways [75]. Similarly, NGAL reduces invasion by suppressing focal adhesion kinase (FAK) activation and inhibits angiogenesis by blocking VEGF production in a model of advanced pancreatic cancer [76]. In human lung carcinoma cells, NGAL might exert a protective role against oxidative stress by inducing the expression of heme oxygenase-1 and superoxide dismutase 1,2 [77]. Three investigations have already analyzed the role of NGAL in multidrug resistance [78,79,80]. While NGAL does not interfere with doxorubicin resistance in breast cancer cells [78], it might contribute to erlotinib (a tyrosine kinase inhibitor of the epidermal growth factor (EGF) receptor) resistance in non-small cell lung cancer cells [80]. In contrast, NGAL could favor the intracellular accumulation of Rhodamine-123 in chronic myeloid leukaemia (CML) and breast cancer cell lines [79]. In all these studies described above, the NGAL receptor involved in the cellular events and the protein’s iron status have not been characterized and could explain NGAL’s divergent effects.

## 5. Possible Roles of the Pro-MMP-9/NGAL Complex

The expression of the pro-MMP-9/NGAL complex often correlates with the aggressive behavior of neoplastic cells and their invasive properties [74,81,82,83]. A few studies suggested that the complex could increase pro-MMP-9’s enzyme activity via an autocatalytic process [83,84,85] thus favoring the invasion of cancer cells through the basement membrane [86]. An *in vitro* study showed that activation of pro-MMP-9 can be mediated by entrapping the remaining *N*-terminal sequence residues of the partially truncated proenzyme into the hydrophobic binding pocket of NGAL [84]. Whether pro-MMP-9 bound to NGAL retains an enzyme activity *in vivo* has to be definitely established. 

Binding of pro-MMP-9 to a gelatin- or type IV collagen-coated surface could lead to reversible activation of MMP-9 via disengagement of the propeptide from the active site [87]. Interaction of hemin or β-hematin with the pro-MMP-9 PEX domain primes MMP-9 activation via an autocatalytic process [88]. Whether a similar mechanism occurs with the pro-MMP-9/NGAL complex remains to be demonstrated. Finally, a growing body of evidence suggests that by binding cell surface receptors, pro-MMP-9 and NGAL can initiate signal transducing events that control tumour cell processes. It is therefore legitimate to suggest that the pro-MMP-9/NGAL complex could interfere with the binding of NGAL and/or pro-MMP-9 to their respective receptors, thus modulating signalling events induced by pro-MMP-9 and/or NGAL (Figure 2).

## 6. Conclusions and Perspectives

The above mentioned data on pro-MMP-9 and NGAL in leukaemia indicate their differential expression between malignant and normal hematopoietic cells. During the initial stages of the leukaemic process, elevated serum levels of both MMP-9 and NGAL are observed in CML patients, while those who respond to treatment with imatinib show a significant decrease in serum NGAL levels. Overexpression of pro-MMP-9 correlates with a poor clinical outcome for patients with AML, ALL and CLL, whereas NGAL expression has not yet been measured in these contexts. The expression of pro-MMP-9 and NGAL has also been detected in other haematological malignancies. Multiple myeloma cells produce pro-MMP-9 and pro-MMP-9/NGAL [89,90,91,92]. In patients with Hodgkin’s and non-Hodgkin’s lymphoma, serum MMP-9 levels are significantly elevated and are associated with poor survival rates [12,93]. It remains to be seen whether the pro-MMP-9/NGAL complex can be detected in the plasma or serum from patients with these hematologic malignancies and whether levels of the complex might be predictive of disease status.

MMP-9 indirectly regulates signalling pathways that control cell growth, survival, invasion and angiogenesis (Figure 2). A growing body of evidence suggests that by binding cell surface receptors (including integrins, CD44, LRP-1/-2 and SLC22A17), pro-MMP-9 and NGAL can directly initiate signal transducing events that control tumour cell processes (Figure 2). The signalling pathways by which these receptors induce cellular responses may be distinct or similar but are thought to rely on the activation of key signalling pathways in tumour cell events. In the normal hematopoietic system, all these receptors are expressed (with different expression profiles) by erythroid, lymphoid and granulocyte/macrophage lineages [7,20,67,94,95]. In contrast to the well-characterized expression patterns of CD44 and integrins in leukaemias [96,97], LRPs and SLC22A17 have not been studied in these diseases and thus require investigation. Whether the pro-MMP-9/NGAL complex, like MMP-9, could display an enzymatic activity and/or influence the signalling actions of pro-MMP-9 and NGAL remains to be unambiguously demonstrated.

Antiproteolytic therapies have sought to target MMP-9s’ catalytic activity and thus inhibit tumor progression [62,98,99]. The failure of MMP-9 inhibitors in phase III clinical trials may be explained by their lack of selectivity towards MMP-9 [62,98,99]. There is now evidence that MMP-9 has complex functions, and that the enzyme inhibitor approach may no longer be sufficient because it does not address pro-MMP-9’s interaction with its “receptors” and the subsequent cell signalling. Hence, novel therapeutic strategies involve newly designed inhibitors, such as peptides that block pro-MMP-9-cell surface interactions and function-blocking anti-MMP-9 antibodies [100,101,102,103]. At present, no specific NGAL inhibitors are available. It remains to be seen whether treatment with specific anti-NGAL or anti-pro-MMP-9/NGAL antibodies might counter the malignant process.

In conclusion, randomized studies are needed for definitely and simultaneously validating pro-MMP-9, NGAL and pro-MMP-9/NGAL as reliable biomarkers in leukaemias and other hematological malignancies. There is also a need for much more work on the triad’s cellular activities in order to develop novel inhibitors for potential use in combination with conventional treatments for hematopoietic as well as solid tumors.
